# Evaluation of a Cetylpyridinium Chloride-, Dipotassium Glycyrrhizinate-, and Tranexamic Acid-based Mouthwash after Implant Placement: A Double-blind Randomised Clinical Trial

**DOI:** 10.3290/j.ohpd.b966793

**Published:** 2021-02-19

**Authors:** Hiromi Taninokuchi, Hidemi Nakata, Yuta Takahashi, Kensuke Inoue, Shohei Kasugai, Shinji Kuroda

**Affiliations:** a Dentist, PhD Candidate, Department of Oral Implantology and Regenerative Dental Medicine, Division of Oral Health Sciences, Graduate School of Medical and Dental Sciences, Tokyo Medical and Dental University, Tokyo, Japan. Idea, experimental design, data collection and analysis, wrote the manuscript.; b Assistant Professor, Department of Oral Implantology and Regenerative Dental Medicine, Division of Oral Health Sciences, Graduate School of Medical and Dental Sciences, Tokyo Medical and Dental University, Tokyo, Japan. Idea, experimental design, data analysis, wrote the manuscript.; c Laboratory Technician, Clinical Laboratory of Dental Hospital, Tokyo Medical and Dental University, Yushima, Tokyo, Japan. Data collection.; d Dentist, PhD Student, Department of Oral Implantology and Regenerative Dental Medicine, Division of Oral Health Sciences, Graduate School of Medical and Dental Sciences, Tokyo Medical and Dental University, Tokyo, Japan. Sequence generation, allocation concealment.; e Professor Emeritus, Department of Oral Implantology and Regenerative Dental Medicine, Division of Oral Health Sciences, Graduate School of Medical and Dental Sciences, Tokyo Medical and Dental University, Tokyo, Japan. Idea, experimental design.; f Junior Associate Professor, Department of Oral Implantology and Regenerative Dental Medicine, Division of Oral Health Sciences, Graduate School of Medical and Dental Sciences, Tokyo Medical and Dental University, Tokyo, Japan. Idea, experimental design.

**Keywords:** antibacterial agents, biofilms, implantology, microbiology, periodontology

## Abstract

**Purpose:**

To evaluate the positive effects of a CPC-, GK2-, and TXA-based (CPC/GK2/TXA) mouthwash after implant placement.

**Materials and Methods:**

Twenty patients (n = 20) who underwent posterior implant-placement surgery were randomly and evenly allocated to the study or the placebo group. After the mouthwash was used 3x/day for 7 to 10 days postoperatively, sutures were analysed by counting the colony-forming units (CFU) for total aerobes, total G [-] anaerobes, total enterobacteria and total *H. influenzae*, followed by Real-Time PCR of bacterial-specific DNAs of *A. actinomycetemcomitans, P. gingivalis, T. forsythia, T. denticola, P. intermedia, P. micra, F. nucleatum, C. rectus,* and *E. corrodens*. In vitro resistance of *P. gingivalis, S. aureus,* and *P. aeruginosa* was analysed. The compatibility of the mouthwash with Straumann SLA implant surfaces was evaluated by scanning electron microscopy (SEM).

**Results:**

Sixteen patients (n = 16) completed the trial. A statistically significantly greater number of CFU was found in the placebo group for almost all species, especially for total G [-] anaerobes. No statistically significant in vitro resistance was found for *P. gingivalis, S. aureus,* and* P. aeruginosa*. SEM revealed no surface alteration after exposure to the mouthwash.

**Conclusion:**

The use of a CPC/GK2/TXA mouthwash inhibited propagation of the bacteria extracted from the post-surgical sutures after implant placement.

In implant dentistry, a profound and thorough oral hygiene routine including the use of mouthwashes is highly recommended, as it influences treatment success and represents a preventive healthcare measure against contamination. Many risk factors are associated with the development of peri-implant diseases, such as peri-implantitis and peri-implant mucositis,^10,34^ but pathogenic biofilm remains a key factor in their initiation and progression.^9,21^

Cetylpyridinium chloride (CPC) is a type of quaternary ammonium compound known for its bactericidal effect.^16,26^ Its mechanism of action consists in the destruction of the bacterial membrane, followed by cytoplasmic leakage.^13,14^ It is widely used in dentistry as a gold-standard antiplaque agent together with chlorhexidine. Nevertheless, it is usually preferred over chlorhexidine because of fewer side effects and similar bactericidal properties.^12^

Dipotassium glycyrrhizinate (GK2) is a dipotassium salt of the glycyrrhetic acid (GA), recognised for its anti-inflammatory properties, and is widely used to prevent hepatic cancer in patients with chronic hepatitis C.^1^ Additionally, recent studies have shown its anti-oxidant properties by inhibiting apoptosis^19^ and possibly inhibiting the NF-κB signaling pathway in rats.^32^ Tranexamic acid (TXA) is an anti-hemorrhagic agent showing satisfactory results in preventing oral bleeding and intraoperative hemorrhage during sinus surgery.^15,33^

Previous studies have clinically evaluated the performance of CPC-based mouthwashes,^8,30^ and even of CPC- and TXA-based mouthwashes.^18^ Nevertheless, there is a paucity of literature available regarding the use of CPC, GK2, and TXA together in the same mouthwash. For this, further RCTs (randomised controlled trials) and in vitro studies are much needed. To the authors’ knowledge, no double-blind RCT with calculation of bacterial CFU nor Real-Time PCR testing has ever been conducted for this kind of mouthwash. In this study, our aim was to evaluate the effectiveness of all three components together in a novel mouthwash through a double-blind RCT, focusing on the bactericidal effect, compatibility with titanium surface, and patient satisfaction.

## Materials and Methods

This parallel study was completed in accordance with the Declaration of Helsinki and was approved by the Ethics Committee at the University Dental Hospital of the Tokyo Medical and Dental University (approval number: D2019-049), registered and accessible in The University Hospital Medical Information Network Center (UMIN-CTR).

### Subjects and Group Allocation

**Fig 1:**
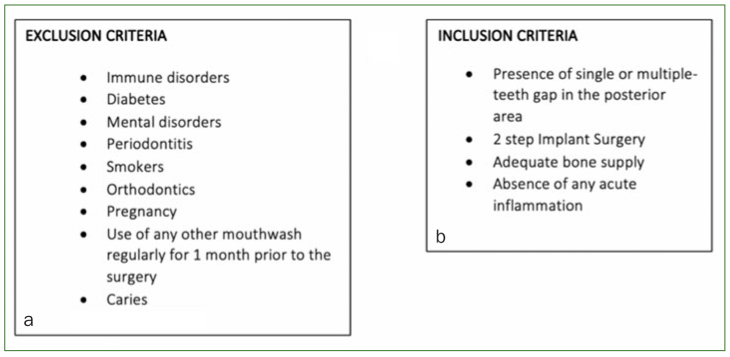
a. Exclusion criteria; b. inclusion criteria. Patients did not take any antibiotics during the pre-surgical period.

**Fig 2:**
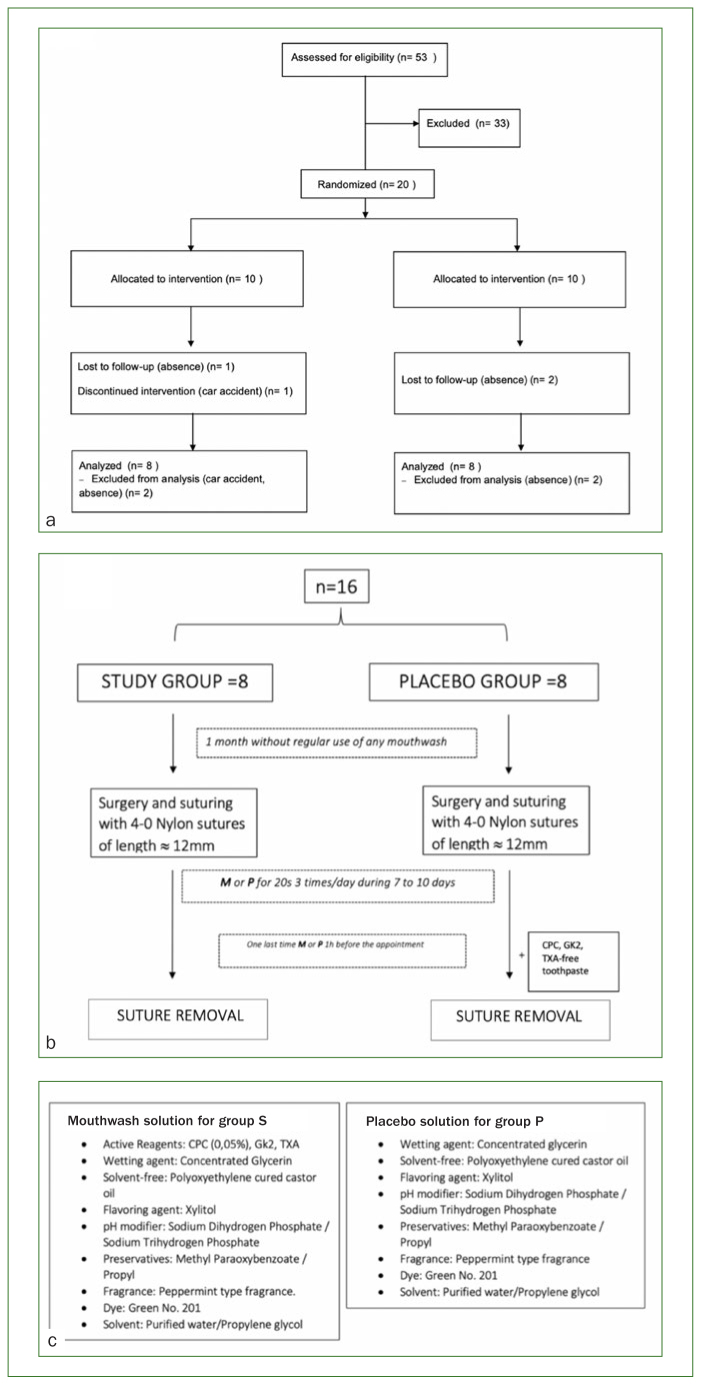
a. Flow diagram of group allocation; b. timepoints of the study until suture removal; c. components of the two solutions used in either group S or group P.

Sample size was defined after reviewing previous literature of similar trials which focused on oral bacteria and mouthwashes.^2,5,25^ In this double-blind study, n = 53 patients were assessed for eligibility among those planning to undergo implant placement surgery at the University Dental Hospital, Tokyo Medical and Dental University, during a determined period of time. Following this, pre-established selection criteria were applied for the selection process (Fig 1): n = 33 patients were excluded, and n = 20 (12 female subjects and 8 male subjects) with a median age of 61) were included after giving their written consent (Fig 2a). All participants were randomly allocated to the study (S) or the placebo (P) group after block randomisation performed by a researcher who was not in contact with any of the participants during any of the clinical phases of this study (KI), so that none of the head clinicians nor the data collectors would know the assignment to interventions. After surgery, patients used 20 ml of the mouthwash solution 3x/day for 20 s until suture removal. In group S, patients utilised a CPC/GK2/TXA mouthwash (Mondamin Pro-care alpha, Earth; Tokyo, Japan), whereas patients in group P utilised a solution without CPC/GK2/TXA but with the same organoleptic properties as the mouthwash used in group S, together with a CPC-, GK2-, and TXA-free toothpaste (Etiquette Lion, Lion; Tokyo, Japan) in order to prevent any cross-reactivity among the components (Figs 2b and 2c).

### Implant Placement Surgery

All surgeries were performed in the Clinical Department of Implantology, University Dental Hospital, Tokyo Medical and Dental University. Surgeries followed the “two-stage dental implant placement” method, where the flap was positioned over the implant.

### Sampling

**Fig 3:**
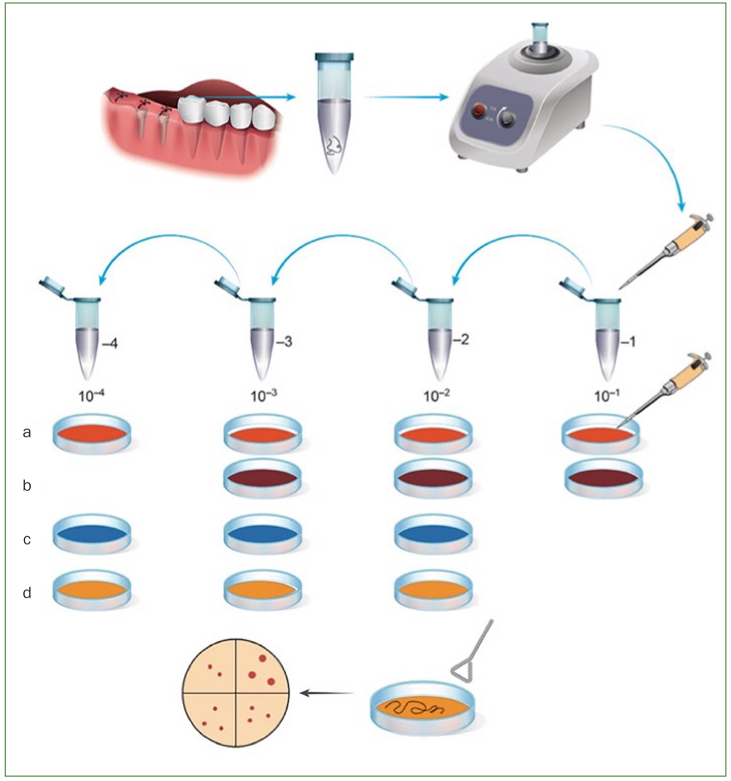
Bacterial culture and CFU counting. Two 4-0 Nylon sutures per patient were collected from the posterior post-surgical regions, then were immersed in sterilised PBS, and the resulting solution was plated onto a. BD Trypticase Soy Agar II with 5% sheep blood; b. PV Brucella HK agar; c. BD Drigalski lactose agar; d. Eiken pourmedia hemophillus agar.

All patients were asked to rinse their mouth one last time 1 h prior to the appointment, and to refrain from eating and drinking afterwards (Fig 2b). Seven to 10 days after implant placement, patients came to the clinic for suture removal. Using sterilised dental instruments, two 4-0 nylon sutures approximately 12 mm long were removed and immersed in 2 ml of sterilised PBS in 15-ml conical sterile polypropylene centrifuge tubes (Nunc, Thermo Fisher Scientific; Waltham, MA, USA) (Fig 3).

### In Vitro Bacterial Culture

Colony forming units (CFU) were counted for total aerobes (TA), total G [-] anaerobes (TGNA), total Enterobacteria (TE) and total *H. influenzae* bacteria (TH). Once collected, all tubes containing the samples were centrifuged for 60 s in order to detach the plaque from the sutures. After this, the sample solution was serially diluted and 100 μl were inoculated onto the respective plates. All plates were incubated at 37°C with a 5% to 10% CO_2_ atmosphere, except those containing TGNA, for which an anaerobic environment was provided by an anaerobic incubator (Hirasawa Works; Tokyo, Japan), and TH, for which a CO_2_-rich environment was created by the candle jar technique for growing capnophiles (Fig 3).

The following media were used for culturing bacteria: a. BD trypticase soy agar II with 5% sheep blood (BD Diagnostics; Franklin Lakes, NJ, USA) for TA; b. PV Brucella HK agar (Kyokuto Pharmaceutical Industrial; Tokyo, Japan) for TGNA; c. BD Drigalski lactose agar (BD Diagnostics) for TE; d. Eiken pourmedia haemophilus agar (Eiken Chemical; Tokyo, Japan) for TH (Fig 3).

### Real-Time PCR Analysis

*Aggregatibacter actinomycetemcomitans* (*A. actinomycetecomitans*), *Porphyromonas gingivalis* (*P. gingivalis*), *Tannerella forsythia* (*T. forsythia*), *Treponema denticola* (*T. denticola*), *Prevotella intermedia (P. intermedia), Parvimonas micra (P. micra), Fusobacterium nucleatum (F. nucleatum), Campylobacter rectus (C. rectus),* and* Eikenella corrodens (E. corrodens)* were analysed by Real-Time PCR. All samples from participating patients were taken following the same protocol, then were analysed using PCR (perio-analyse, Pierre Fabre Medicament; Paris, France).

### In Vitro Resistance

Resistance of *Porphyromonas gingivalis* (ATCC 33277) (*P. gingivalis*), *Staphylococcus aureus* subsp. *aureus* KWIK-STIK (ATCC 29213) (*S. aureus*), and *Pseudomonas aeruginosa* KWIK-STIK (ATCC 27853) (*P. aeruginosa*) to a CPC/GK2/TXA mouthwash was evaluated in vitro.

Bacterial suspensions were adjusted separately to be equivalent to the turbidity of 10^5^ McFarland standards, then a 100x diluted solution was created by adding 0.1 ml of the bacterial suspension to 9.9 ml of either a CPC/GK2/TXA solution or a solution without CPC/GK2/TXA. Samples from each solution were plated after 1 min of exposure in the tubes. Solutions containing *S. aureus* and *P. aeruginosa* were plated onto BD Trypticase Soy Agar II with 5% sheep blood (BD Diagnostics), and results were analysed after 48 h of aerobic incubation. For *P. gingivalis*, bacterial solutions were plated on PV Brucella HK agar (Kyokuto Pharmaceutical Industrial) and results were analysed after 96 h of anaerobic incubation using AnaeroPacks (Mitsubishi Gas Chemical; Tokyo, Japan).

A quantitative analysis was performed with ImageJ (version 2.0.0, National Institutes of Health; Bethesda, MD, USA) to measure the amount of surface colonisation by bacteria after transforming all images to RGB Stack and setting the corresponding color threshold.

### Visual Analogue Scale (VAS)

A VAS was applied in group S to analysing potential detrimental effects and subjective features of a CPC/GK2/TXA mouthwash at the end of the study. Factors evaluated included irritating sensation, organoleptic properties, and overall satisfaction.

### Statistical Analysis

The p-value was evaluated with a Mann-Whitney test and an unpaired F-test to compare variances (Prism8 for MacOS). Results with p < 0.05 were considered statistically significant. p-values ≥ 0.05 were not significant (ns).

### SEM Analysis

Two Straumann SLA implants were immersed either in the mouthwash solution or the placebo solution for 48 h. Results were evaluated using SEM (Horiba S-817XL; Kyoto, Japan) at magnifications of 5000X and 1000X.

## Results

During the study, three patients were lost to follow-up (n = 3) and one discontinued the intervention due to a car accident (n = 1). Sixteen volunteers (n = 11 women, n = 5 men) with a median age of 63.3 were analysed in this study (Fig 2a).

### Bacterial CFU

**Fig 4:**
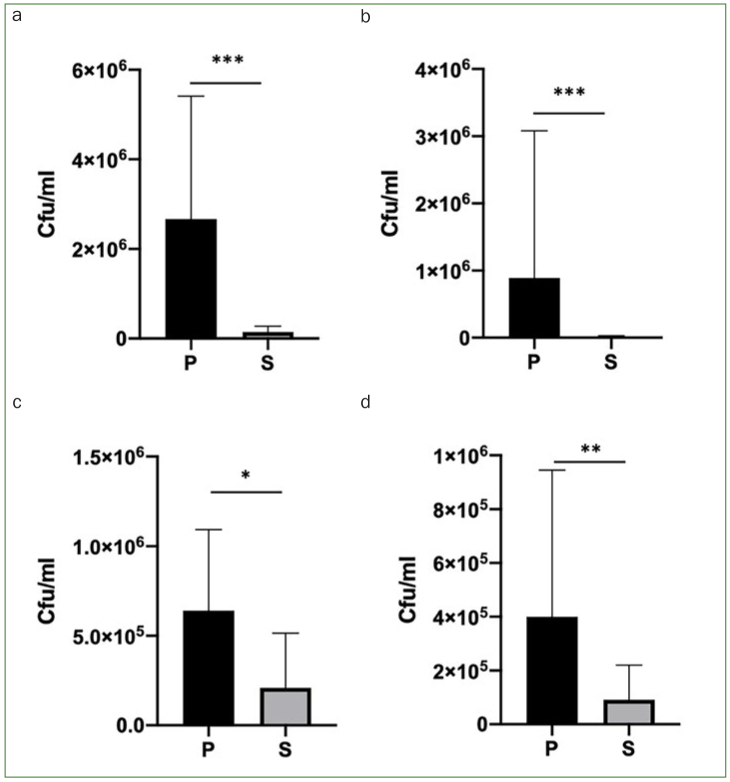
Bacterial counts in CFU/ml of post-surgical sutures. Mean with SD of CFU for total aerobic bacteria (a), total G [-] anaerobic (b), total enterobacteria (c), and total *H. influenzae* (d) in S and P (n = 8). ***p = 0.0001 to 0.001; **p = 0.001 to 0.01; *p = 0.01 to 0.05; ns: p ≥ 0.05.

Bacterial CFU were statistically significantly higher for group P than for group S. The number of bacteria in group P was statistically significantly higher for TA, TGNA, TE, and TH (p<0.05) (Fig 4).

### Bacterial Real-Time PCR

**Fig 5:**
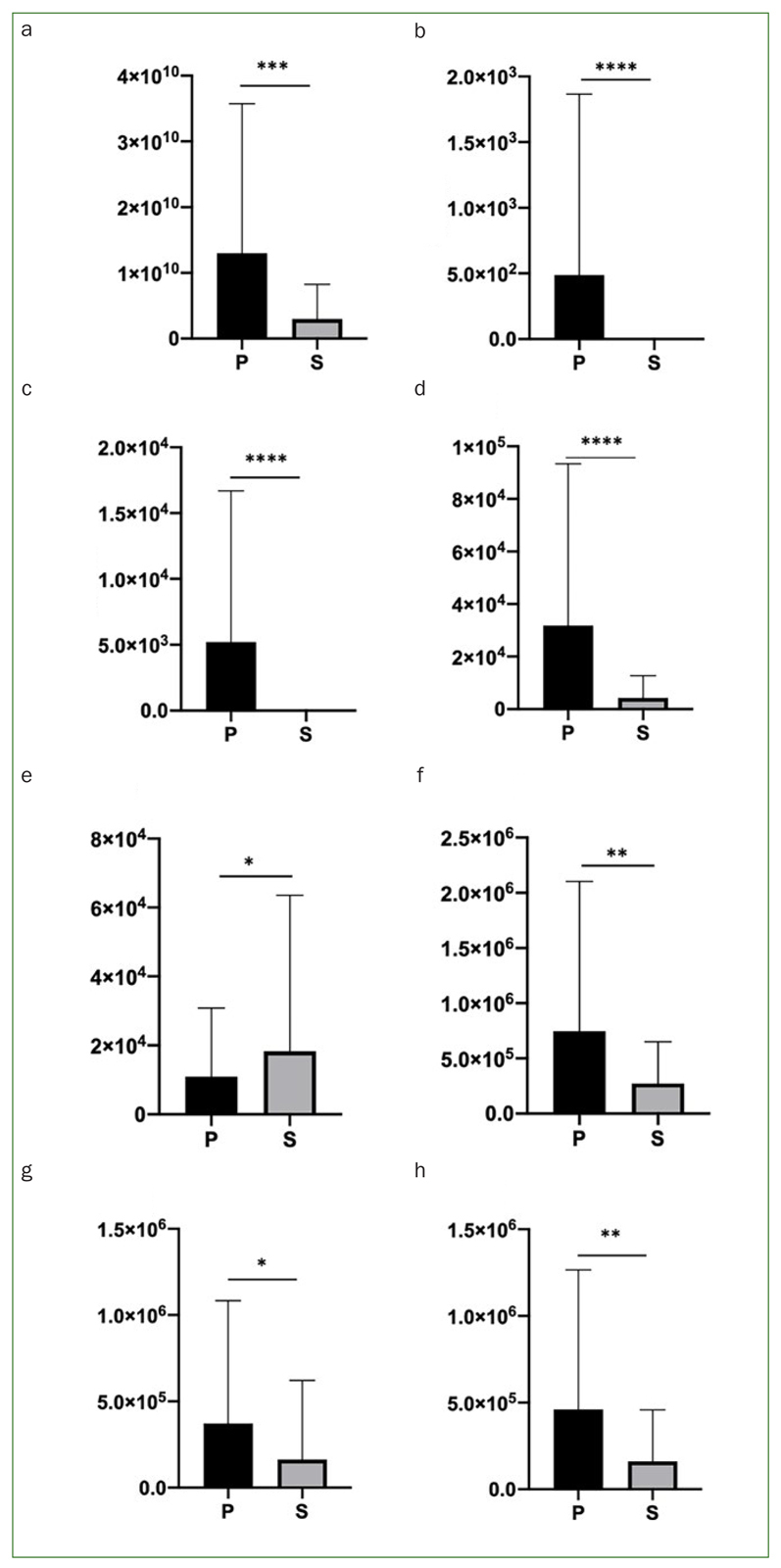
Bacterial amounts on post-surgical sutures. Mean with SD of Real-Time PCR results for total bacteria (a), *P. gingivalis* (b), *T. forsythia* (c), *P. intermedia* (d), *P. micra* (e), *F. nucleatum* (f), *C. rectus* (g), and *E. corrodens* (h) in S and P (n = 8). ****p < 0.0001; ***p = 0.0001 to 0.001; **p = 0.001 to 0.01; *p = 0.01 to 0.05; ns: p ≥ 0.05.

Bacterial Real-Time PCR results showed that the overall amount of bacteria was statistically significantly higher for group P than for group S (Fig 5). These results showed a lower concentration of bacteria for group S than for group P in most cases, except for *P. micra* (Fig 5e). No *A. actinomycetemcomitans* or *T. denticola* were observed in any of the samples. On the other hand, *P. ginvivalis* or *T. forsythia* were present only in group P, not in group S (Figs 5b and 5c).

### Bactericidal Effect of a CPC/GK2/TXA Solution on *P. gingivalis, S. aureus*, and *P. aeruginosa*

**Fig 6:**
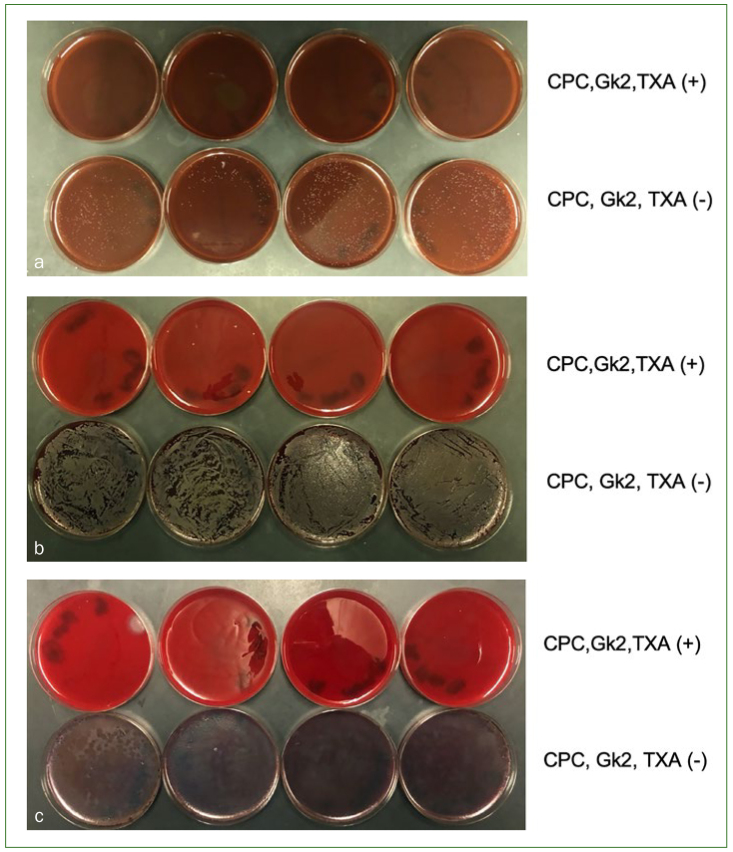
In vitro resistance of *P. gingivalis, S. aureus*, and *P. aeruginosa*. Surface colonisation in vitro of *P. gingivalis* (a), *S. aureus* (b), and *P. aeruginosa* (c), after exposure to either a CPC/GK2/TXA solution [CPC, GK2, TXA (+)] or a solution without CPC/GK2/TXA [CPC, GK2, TXA (-)].

Surface colonisation by *P. gingivalis, S. aureus,* and *P. aeruginosa* was inhibited by the CPC/GK2/TXA solution (Fig 6a to 6c). The bactericidal effect of CPC/GK2/ TXA was statistically significant for *S. aureus* and *P. aeruginosa,* and was complete for *P. gingivalis,* which in this case did not show black pigmentation correspondingly to the manufacturer’s instructions of ATCC 33277 where it is explained that black pigmentations appear after 5 days of incubation.

### Visual Analogue Scale (VAS)

VAS results showed overall satisfaction among patients in group S. Restricted to group S, 87% of the patients qualified the mouthwash as ‘not at all irritating’. Regarding organoleptic properties, 87% of the patients described the flavour as ‘not at all unpleasant’. 87% confirmed their willingness to continue using this mouthwash solution (Figs 8 and 9).

**Fig 7:**
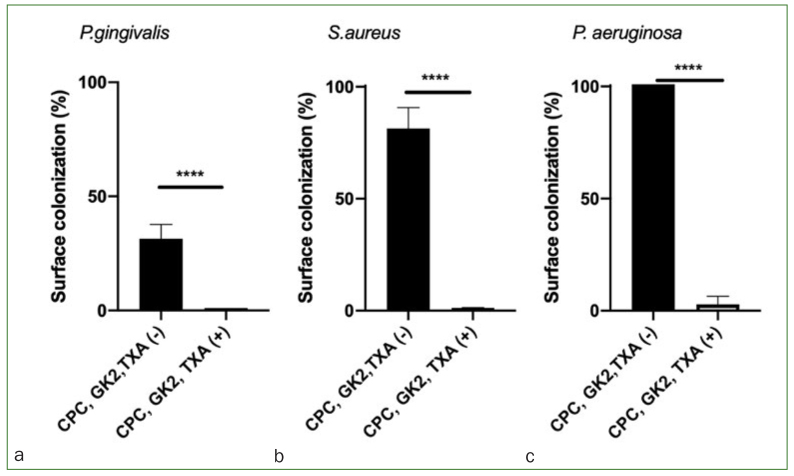
Surface colonisation of *P. gingivalis* (a), *S. aureus* (b), and *P. aeruginosa* (c), after exposure to either a CPC/GK2/TXA solution [CPC, GK2, TXA (+)] or a solution without CPC/GK2/TXA [CPC, GK2, TXA (-)]. Values are expressed as (x+1) on the y-axis. ****p<0.0001.

**Fig 8:**
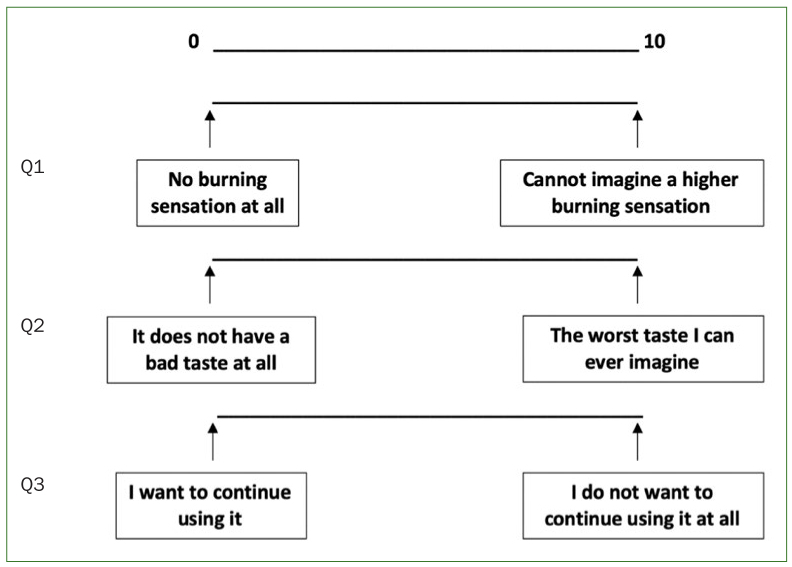
Visual analogue scale (VAS). All patients belonging to group S answered a VAS regarding irritating sensation, organoleptic properties, and overall satisfaction.

**Fig 9:**
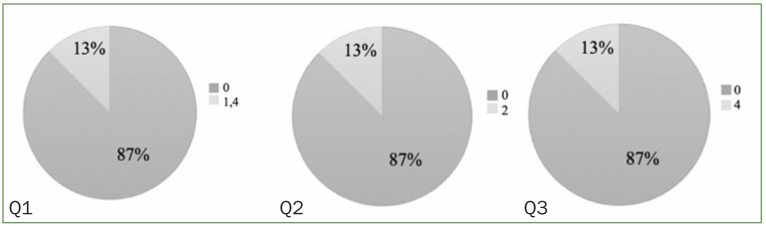
VAS results from patients belonging to group S. For Q1, 87% of patients indicated a VAS score of 0 for irritating sensation and 13% indicated a score of 1.4. For Q2, 87% of patients indicated a VAS score of 0 regarding organoleptic properties and 13% gave a score of 2. For Q3, 87% of patients want to continue the use of the mouthwash.

### Surface Alteration Evaluated by SEM

**Fig 10:**
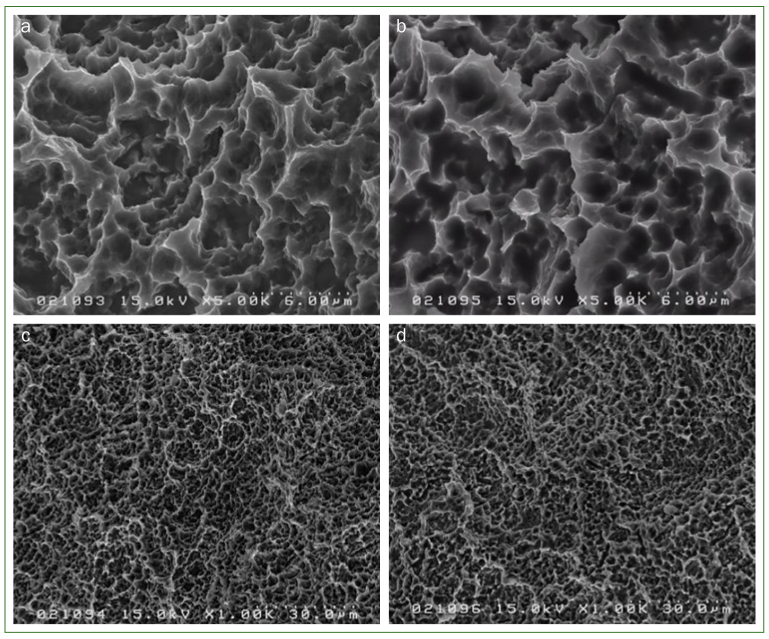
SEM images of the titanium surface. No surface alteration was observed after immersion of the titanium surface in the mouthwash solution. SEM images at 5000X magnification after 48 h of titanium implant immersion in either a. CPC/GK2/TXA solution or b. solution without CPC/GK2/TXA. SEM results at 1000X magnification after 48 h of titanium implant immersion in either c. CPC/GK2/TXA solution or d. solution without CPC/GK2/TXA.

No surface alteration of SLA implants was observed after 48 h of immersion. SEM images (5000X and 1000X) showed no surface alteration after exposure to CPC/GK2/TXA. Similarly, no corroded tubercles were observed on the metal surface of either implant exposed to the mouthwash solution or to the placebo solution (Fig 10).

## Discussion

As the mechanical cleaning of the post-surgical area requires careful attention, mouthwashes with anti-bacterial, pro-healing and regenerative properties might be useful to chemically prevent the formation of plaque and enhance the healing of soft tissues.

In previous studies, *A. actinomycetemcomitans* has been detected in patients with aggressive periodontitis and peri-implantitis.^28,29^ However, some controversy exist regarding the microorganisms involved in peri-implantitis.^3,31^ Since no patients with periodontal disease were included in this study, *A. actinomycetemcomitans* and *T. denticola* were not detected in any of the samples. These results confirm the low probability of finding this type of bacteria in patients without periodontal disease or peri-implantitis. However, it must be mentioned that even though no patients with periodontitis or peri-implantitis were included in this study, bacteria such as *P. gingivalis* and *T. forsythia* were present on the nylon sutures (only in group P). Nylon sutures are generally recommended to clinicians for their superiority in terms of anti-plaque properties compared to other types of sutures. Thus, the possible colonisation of the tissue surrounding the newly placed implant by dangerous pathogenic bacteria emphasises the importance of plaque control during this critical phase.

On the other hand, *F. nucleatum* which works as a bridge between early and late colonizers, such as *A. actinomycetemcomitans, T. denticola, P. gingivalis,* among others,^17^ was statistically significantly higher in group P. Previous studies showed that gram negative bacteria are sometimes resistant to quaternary ammonium compounds.^24^ Nevertheless, our results suggested that in vivo gram-negative bacteria were also sensitive to CPC, and that the overall amount of bacteria in group P was statistically significantly higher than in group S, including almost all species, including aerobic and anaerobic bacteria (Figs 4 and 5). We were also able to prove the bactericidal effect of the mouthwash used in vitro in this study on highly pathogenic* P. gingivalis, S. aureus* and *P. aeruginosa*.^7,11,20,27^ However, the fact that no permanent surveillance or control regarding conformity with instructions was feasible and that possible subjective variations in normal flora from patient to patient might exist, should be mentioned as limitations of this study, together with the fact that healing and inflammation level were not measured in this study. Further studies should be performed to evaluate the anti-inflammatory and anti-hemorrhagic properties of this mouthwash.

Depending on the components, particular toothpastes or mouthwashes are recommended to implant-treated patients because of their lack of corrosiveness.^6,23^ Since no surface alterations, e.g. corrosion, were observed by SEM, the use of these components in mouthwashes would be acceptable to implant-treated patients. On the other hand, patients with xerostomia or burning mouth syndrome are statistically significantly more predisposed to oral infections than patients with a normal salivary flow rate.^4,22^ Since in this study, 87% of the patients described the mouthwash solution as “not at all irritating”, it could therefore be particularly recommended to any patient with a pronounced sensitivity to alcohol-based mouthwashes, which can be highly irritating (Figs 8 and 9).

Additionally, healing was clinically evaluated in all patients; it was visibly improved in group S compared to group P, probably due to the presence of GK2 and TXA. However, further studies are required to confirm pro-healing properties and elucidate details about CPC specificity in the field of peri-implant plaque control.

## Conclusion

The use of a CPC/GK2/TXA mouthwash predominantly inhibited the activity of aerobic and anaerobic bacteria attached to post-surgical sutures after implant placement.

## Clinical relevance:

This is an evaluation of a novel mouthwash containing CPC, GK2, and TXA with bactericidal and antihemorrhagic properties in dental patients.
